# Detection of Crimean Congo haemorrhagic fever virus in North-eastern Senegal, Bokidiawé 2019

**DOI:** 10.1080/22221751.2020.1847605

**Published:** 2020-11-20

**Authors:** Idrissa Dieng, Mamadou Aliou Barry, Moussa Moise Diagne, Boly Diop, Mamadou Ndiaye, Martin Faye, Marie Henriette Dior Ndione, Mame Malick Dieng, Abdoulaye Bousso, Gamou Fall, Cheikh Loucoubar, Amadou Alpha Sall, Oumar Faye, Ousmane Faye

**Affiliations:** a Département de Virologie, Institut Pasteur de Dakar, Dakar, Senegal; b Prevention Department Ministry of Health, Dakar, Senegal

**Keywords:** CCHF, Genotype III, Senegal, North-East, Mauritania, 2019

## Abstract

We diagnosed a human case of Crimean Congo hemorrhagic fever (CCHF) in Bokidiawe (North-eastern Senegal), 2019. The phylogenetic analysis revealed that the isolate belongs to genotype III and is closely related to a strain reported in Mauritania in 1984 and Spain in 2016. Distribution area of CCHF in Senegal is progressively increasing.

Crimean Congo haemorrhagic fever (CCHF) is an acute, viral, zoonotic disease circulating in Africa, Asia and Europe where the primary vectors Hyalomma ticks have a wide distribution [1]. In nature it is transmitted through an enzootic cycle between a various number of mammals and birds as well as Ixodid ticks [1]. The etiologic virus belongs to the family Nairoviridae and was first isolated in Congo in 1956 while the disease was firstly described in the Crimea in 1944, leading to the name Crimean Congo haemorrhagic fever virus (CCHFV) in 1969 [2]. CCHFV persists throughout the life cycle of ticks, making them reservoirs of infection for long periods without vertebrate hosts [2].

In humans, CCHFV can be transmitted by infected ticks bite, by direct contact with blood or infected tissues from animals [1] and human-to-human transmission through virus-containing-body-fluids, mainly in a nosocomial context [3]. CCHF shows a spectrum of severity ranging from mild non-specific febrile syndrome to vascular leakage, multi-organ failure, shock and haemorrhagic signs [1]. CCHFV infection may be subclinical or asymptomatic in some people as highlighted by its high seroprevalence in some European populations [2].

CCHFV has a negative tripartite RNA genome with three segments namely S (small), M (medium), L (large) [2] Based on the S segment, CCHFV shows a large diversity worldwide with 6 distinct lineages [4]. A geographic structuration of described genotypes was reported Indeed the genotype V and VI are circulating in Europe: the genotype IV in Asia while genotypes I, II, III are described in Africa [1]. Despite this established distribution, strains can move and be dispersed between geographic regions through livestock movements, birds migration and human travel [5].

In West Africa, the first human cases of haemorrhagic fever linked to CCHF were described in Mauritania in 1983 [6]. In Senegal, CCHF has been found in various locations through the country since 1969 [7]. In the village of Bandia, a human prevalence of 3,2% was previously reported in a study conducted from 1986 to 1989 [7]. In the same area, the virus was isolated from ticks between 1989 and 1992 [8]. Several other isolates were obtained from Hyalomma marginatum rufipes nymphs collected on birds [8]. A new case was also reported from a young shepherd in 2003 in a rural district 60 km far from Dakar, the capital, followed by two other cases detected from French tourists in 2004 [9].

## The study

On 4 September 2019, a 47-year old woman living in a village in Douga (Matam region: North-eastern Senegal, Appendix Figure) was received at the health care of Bokidiawe sentinel site with a pain syndrome onset the day before. She presented with headache, myalgia, retro-orbital pain and a febrile syndrome. The onset of the disease was 3 September 2019. Without malaria infection (malaria RDT negative), hypothesis of a suspected arbovirus infection was mentioned then a blood sample was sent to the Institut Pasteur de Dakar (IPD) on 6 September 2019 as part of ongoing Syndromic Sentinel Surveillance network in Senegal (4S network) [10]. The patient finally recovered without complications.

**Figure 1.**
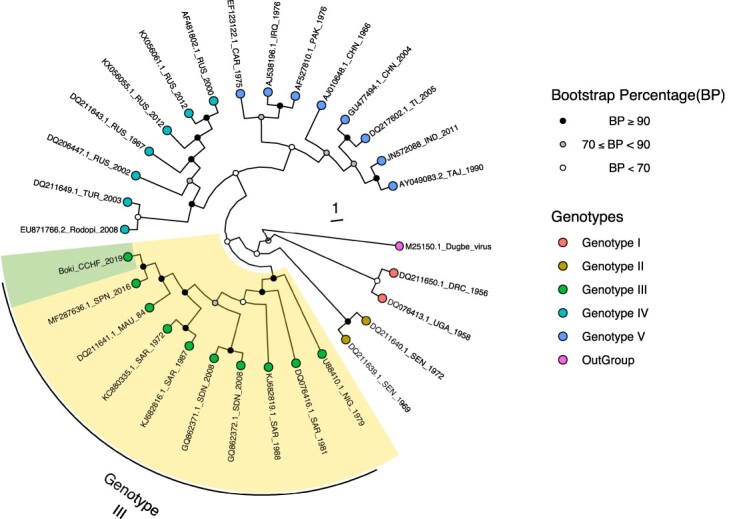
Phylogenetic tree based on partial S segment (850 bp) of CCHFV. The multiple sequence alignment was obtained by using Mafft [12] and the phylogenetic tree was constructed using the maximum likelihood method implemented in IQ-TREE [13]. 1000 replicates of the sequences data were used for the robustess. The tree include 5 genotypes as described by Lukashev et al. [11]. Bootstrap confidence cut off are shown at each node. The strain reported during this study belonging to genotype III is coloured in green and indicated by (*) sign.

At the IPD, viral RNA was extracted from the patient serum sample using the QIAamp viral RNA Mini kit (QIAGEN, Hilden, Germany) and screened for some several arboviruses and heamorrhagic fever viruses by real time RT–PCR from which only CCHF gave a positive result. For the genetic characterization of the strain, the full S segment was amplified using published overlapping two set of primers [11] with Transcriptor One-Step RT–PCR kit (Roche, Germany). PCR was carried out using the following cycle profile: 95°C– 5 min; 10 touchdown cycles (95°C 15″– 65°C to 55°C 20″–72°C 1 min); 40 amplification cycles (95°C 15″– 55°C 20″–72°C 1 min); 72°C– 5 min. PCR products were sequenced in both directions by Sanger sequencing. The obtained sequences were assembled and merged using online tools (emboss revseq: http://www.bioinformatics.nl/cgi-bin/emboss/revseq and emboss merger: http://www.bioinformatics.nl/cgi-bin/emboss/merger). Only sequence obtained with the first primer set with a size of 850 bp had good quality, so further molecular characterization was performed using this partial S segment aligned with representative available CCHFV sequences of known genotypes from GenBank. The alignment was performed using Mafft [12] and a ML phylogenetic tree build with iQ-TREE [13], 1000 replicates for bootstrapping were applied. The nucleotide sequence of the new CCHFV showed respectively 99,91% and 99,27% identity with a strain isolated in Mauritania in 1984 (DQ211641) and an isolate from Spain in 2016 (MF287636), respectively and clustered within the genotype III namely Africa 3 (Figure 1). The high similarity with the Mauritanian isolate supports a possible introduction to Senegal from Mauritania. Interestingly, the genotype III is known to be endemic in Mauritania, South Africa, Sudan and Nigeria [5]. Additionally, the WHO reported in July 2019 one new CCHF case from Kithat in the Wilaya of Guidimakha in the Senegalese-Mauritanian border (WHO, 2019). This combined with the detection of a closely related strain in Spain in 2016 [4] supports the hypothesis that the virus can be introduced into Europe by infected ticks transported from Africa and by migratory birds to neighbouring countries via the trade of livestock [2].

## Conclusions

We report an autochthonous CCHF case detected in 2019 from Bokidiawe, Northeastern Senegal. Sequencing of a part of S segment showed that the current strain belongs to the genotype III that usually circulates in Mauritania [4]. This point supports a possible introduction to Senegal from Mauritania. Previous studies showed that CCHF appears to be endemic in Mbour area as 3 human cases were diagnosed in 2003 [14] and 2004 while 27 CCHFV strains were recovered from ticks and one goat between 1989 and 1996 [15]. Otherwise, four other imported cases from Mauritania and an autochthonous one from Fatick, region at the border with Gambia (more and less 100 km away from Mbour), were also detected in 2017 (unpublished data), suggesting a shift of this transect to the centre or the south of the country. Moreover, livestock has also an important role in the maintenance and transmission of CCHFV with asymptomatic viremia lasting up to 7–15 days, permitting migrations over long distances of infected ruminant populations. Conditions for upsurge of CCHF case is then complex, mixing climatic, abiotic and biotic factors [2].

It is therefore relevant to conduct studies from north to south of the country by investigating humans, ticks and animals in order to better understand the dynamics of CCHFV circulation in Senegal. Additionally, this new case calls for collaborative interventions, disease control and improved surveillance between Senegal and Mauritania in order to reduce the risk of spreading. Healthcare workers should also be advised about the potential CCHFV circulation in this region and to consider it in case of clinical picture linked to arboviral infection or haemorrhagic fever.

Since recent study in Sudan highlighted the fact that CCHFV lineages are not restricted to defined geographic areas [5] and that possible genetic reassortments play an important role in generating diversity [2] and thus impacting the virulence, a full genome characterization of the new strain and further analysis is needed in order to understand its dispersal pattern.

## Supplementary Material

CCHF_Map_final.png
