# Supplementary figures and images for: Detection of Crimean Congo haemorrhagic fever virus in North-eastern Senegal, Bokidiawé 2019
Source: Emerg Microbes Infect. 2020 Nov 20;9(1):2485–7. doi: 10.1080/22221751.2020.1847605 (PMC7717587; doi:10.1080/22221751.2020.1847605)

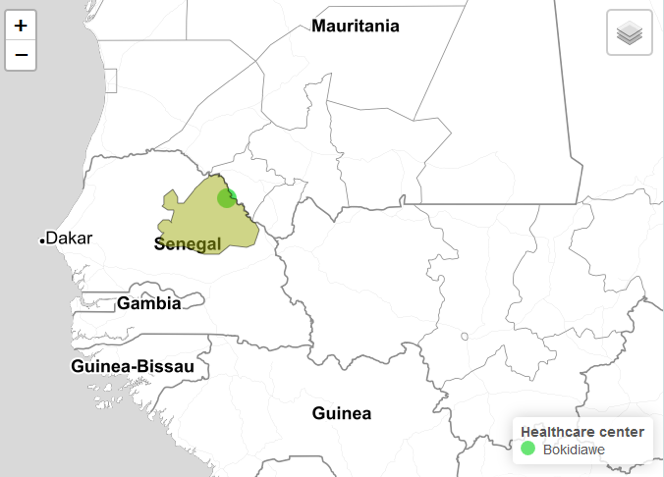

Supplement: CCHF_Map_final.png [file TEMI_A_1847605_SM6362.png]
